# Disinfection of 3D-printed surgical guides using virgin coconut oil (in vitro study)

**DOI:** 10.1186/s12903-023-03092-x

**Published:** 2023-06-10

**Authors:** Rania T. Khalil, Ahmed Alshimy, Eglal Elsherbini, Mervat E. Abd-Ellah

**Affiliations:** 1 Department of Prosthodontics, Faculty of Dentistry, Alexandria University, Alexandria, Egypt; 2 Department of Microbiology, Medical Research Institute, Alexandria University, Alexandria, Egypt

**Keywords:** Disinfection, 3D-printing, Surgical guides, Virgin coconut oil

## Abstract

**Background/objective:**

Disinfection of a 3D-printed surgical guide is of utmost importance as it comes into contact with hard and soft tissue during implant placement so it poses a potential risk of pathogenic transmission. Methods used for disinfection in the surgical field should be reliable, practical, and safe for the instruments and the patients. The objectives of this study were to compare the antimicrobial potential of 100% Virgin Coconut Oil, 2% Glutaraldehyde, and 70% Ethyl Alcohol used to decontaminate 3D-printed surgical guides.

**Materials and methods:**

Thirty identical surgical guides were printed and cut into two halves (N = 60). Both halves were then contaminated with a defined amount of human saliva samples (2 ml). The first half (n = 30) was sub-grouped into three study groups which were immersed in one of the three disinfectants for 20 min as follows; group VCO was immersed in 100% Virgin Coconut Oil, group GA was immersed in 2% Glutaraldehyde, and group EA was immersed in 70% Ethyl Alcohol. The second half (n* = 30) was sub-grouped into three control groups which were immersed in sterile distilled water as follows group VCO*, group GA*, and group EA*. The microbial count was expressed as colony-forming units per plate and the comparison of the antimicrobial potential of the three tested disinfectants between the three study and three control groups was done using the One-Way ANOVA test.

**Results:**

The culture results of three study groups revealed no bacterial growth with the highest % of reduction in the mean microbial count of the oral microorganisms (about100%) and an uncountable bacterial growth was shown between the three control groups (more than 100 CFU/plate) representing the baseline of the oral microorganisms. Therefore; statistically significant differences were found between the three control and three study groups (*P* < .001).

**Conclusion:**

The antimicrobial potential of Virgin Coconut Oil was comparable and equivalent to Glutaraldehyde and Ethyl Alcohol with a significant inhibitory action against oral pathogens.

## Introduction

Prosthodontics has been cited as one of the dental specialties that most neglect infection control guidelines where the dental office-prosthesis laboratory connection represents a tremendous potential for infection pathway if effective disinfection procedures are not performed properly [1]. Cross-infection can be carried directly by blood or saliva and indirectly by contaminated equipment, surfaces, and airway which could transmit infectious diseases such as Human Immunodeficiency Virus (HIV), Hepatitis-B virus, Pneumonia, and Tuberculosis, thus contamination of the dental environment creates a great risk to patients and healthcare workers [2]. Moreover, the global outbreak of coronavirus disease (COVID-19) caused by the novel severe acute respiratory syndrome coronavirus 2 (SARS-CoV-2) made infection control an essential practice for dental clinics and a crucial protocol for preserving the practice’s integrity [3, 4]. Currently, one of the growing technologies that lead to the birth of computer-guided surgery which is changing the manufacturing industry is additive manufacturing, rapid prototyping, or three-dimensional (3D) printing surgical guide, this medical device that aids in selecting the precise site for implant placement and minimizes the operators’ risk of error [5]. Surgical guide is not only known to be porous and has irregularities and crevices which may have a marked effect on microbial adherence and biofilm formation, but also, it comes in contact with the blood, bone, and oral fluids of the patient during surgical procedures and, causes serious infections if it is contaminated by any extraoral pathogens [6].

Accordingly, the United States Food and Drug Administration (FDA), Centers for Disease Control and Prevention (CDC), and the American Dental Association (ADA) categorized the surgical guide as a critical item that penetrates soft tissues or bones and has to be sterilized by steam heat. However, it was claimed that owing to the thermosensitivity of the surgical guide, it should not be sterilized but immersed in effective disinfecting solutions [7]. The existing disinfecting solutions, such as 2% Glutaraldehyde (GA) and 70% Ethyl Alcohol (EA), are potentially hazardous [8]. GA vapor caused serious adverse health effects such as asthma, rhinitis, and, bronchial hyperresponsiveness [9]. Although EA had substantial antimicrobial properties, it evaporated rapidly making extended exposure time difficult to achieve unless the items were immersed [10]. Additionally, certain pathogen populations were becoming more tolerant to EA exposure thus, the use of different antibacterial products in the clinical setting was highly suggested [11].

Subsequently, the emergence of antimicrobial resistance had become a frequent issue for practitioners therefore, researchers had significantly attracted attention to exploring novel antimicrobial compounds utilizing natural products of plant origin [12]. Nowadays, many herbs derived ingredients are being used as antiseptics, such as Cocos nucifera or “Virgin Coconut Oil’’ (VCO) which can be exceptionally advantageous, as it possessed antibacterial, antiviral, antioxidant, antifungal, and antiprotozoal characteristics towards a broad range of microorganisms [13, 14]. VCO is different from most other dietary oils because the predominant composition is a medium-chain fatty acid (MCFA), whereas, in the majority of other oils, the basic building blocks are almost entirely long-chain fatty acids [15]. In this regard, bactericidal activity could be decreased by increasing the chain length of the fatty acids and influencing their physical and chemical properties [16]. Notably, VCO is produced.from fresh coconut without the application of heat, which does not lead to alterationof the nature of the oil and its important content can be preserved [17]. The Medium Chain Triglyceride (MCT) content in VCO is lauric acid followed by myristic acid, palmitic acid, capric acid, caprylic acid, oleic acid, stearic acid, linoleic acid, and caproic acid, as shown in Table 1 [12, 18]. Consequently, VCO contains 92% saturated fatty acids, approximately 50% of which is lauric acids that can prevent infection and excessive cell damage due to activation of TGF-β cytokines which stimulate fibronectin in fibrin clots formation, then become the framework for re-epithelialization and accelerate the healing process [19]. Nevertheless, lauric acid is being formed into monolaurin in the human body, which is demonstrated to be an active antimicrobial monoglyceride [20]. It produces highly ordered membranes, which is thought to disrupt membrane function by affecting signal transduction due to blockage of promoters, uncoupling of energy systems, altered respiration state, and amino acid uptake Furthermore, it could solubilize lipids and phospholipids from bacterial cell membranes causing disintegration, inhibiting pathogen maturation, and preventing its binding to host cells [21]. This leads to the destruction of the cell membrane of DNA and RNA viruses such as *HIV, Herpes*, *Cytomegalovirus, Influenza*, various pathogenic bacteria such as *Listeria monocytogene*, and protozoa such as *Giardia lamblia *[22, 23]. Moreover, the alkalis in the saliva react with VCO resulting in saponification and the formation of a soap-like substance which reduces the adhesion of plaque. Hence, lauric acid reacts with salivary sodium hydroxide forming sodium laureate, the main constituent of soap which might be responsible for the cleansing action and decreased plaque accumulation [24]. Additionally, the acidic pH nature of VCO between 2.52 and 4.38 was considered to be an important attribute of its microbial inhibitory action against many gram-positive and gram-negative organisms such *as Escherichia vulneris, Enterococcer species, Helicobacter pylori, Staphylococcus aureus,* and *Streptococcus mutans* [25–28]. Remarkably, its glucolipid component, sucrose monolaurate has an anti-cariogenic property due to reduced glycolysis and sucrose oxidation and its viscous nature promotes lubrication, thereby inhibiting adhesion of bacteria or its by-products on the mucosal tissues [29]. It is worth noting that VCO has antipyretic and analgesic effects by stimulating the new blood vessels formation and by lowering the surface area of the wound., thus it has a miraculous healing power that acts as a natural antibiotic and also helps modulate immunity [30]. Few published research are reported about the effect of VCO as a disinfectant used for decontamination of 3D printed surgical guides. Thus, this study aimed to compare the antimicrobial potential of 100% VCO, 2%GA, and 70% EA used to decontaminate 3D-printed surgical guides. The null hypothesis was that no significant differences would be found in the antimicrobial potential of 100% of VCO, compared to 2% GA and 70% EA.

**Table 1:** The distribution of the total fatty acid content of VCO [29]

Common name	Carbon number	Composition (%)
Caproic acid	C 6:0	0.80–0.95
Caprylic acid	C 8:0	8.00–9.00
Capric acid	C 10:0	47.00–50.00
Lauric acid	C 12:0	17.00–18.50
Myristic acid	C 14:0	7.50–9.50
Palmitic acid	C 16:0	ND
Palmitoleic acid	C 16:1	2.50–3.50
Stearic acid	C 18:0	4.50–6.00
Oleic acid	C 18:1	0.70–1.50
Linoleic acid	C 18:2	ND
Linolenic acid	C 18:3	0.80–0.95

*ND* Non-detectable

## Materials and methods

### Study setting

This in vitro comparative study was held at the Department of Prosthodontics, Faculty of Dentistry, and the Department of Microbiology, Medical Research Institute, Alexandria University. Prior to commencement, all the methods were approved by the Institutional Review Board (IRB) of Research Ethics Committee at the Faculty of Dentistry, Alexandria University, Egypt (IRB 00010556– IORG 0008839) after ensuring that all methods are in accordance with the Helsinki declaration.

### Sample size calculation

The minimal sample size was calculated based on a previous study aimed to assess the antimicrobial effectiveness of 100% VCO, 2% GA, and 70% EA disinfectants used to decontaminate 3D-printed surgical guides. Khalil et al., [8] concluded that antimicrobial effectiveness was the same between the three tested disinfectants without showing any microbial growth. Based on their results, adopting a power of 80% to detect a standardized effect size in the dimensional accuracy (d = 0.9102) (large-sized standardized effect size), and level of significance 95% (alpha = 0.05), the minimum required sample size was found to be 60 surgical guides (30 surgical guides cut into two halves, number of groups per each half = 3, and number of surgical guides per group = 10 [31]. Any error in the procedure that may lead to the loss of any sample (guide) was compensated by replacing the lost guide with a new one to maintain the required minimum sample size of 60 and to control for attrition (withdrawal) bias [32].

### Study design

**Fig. 1:**
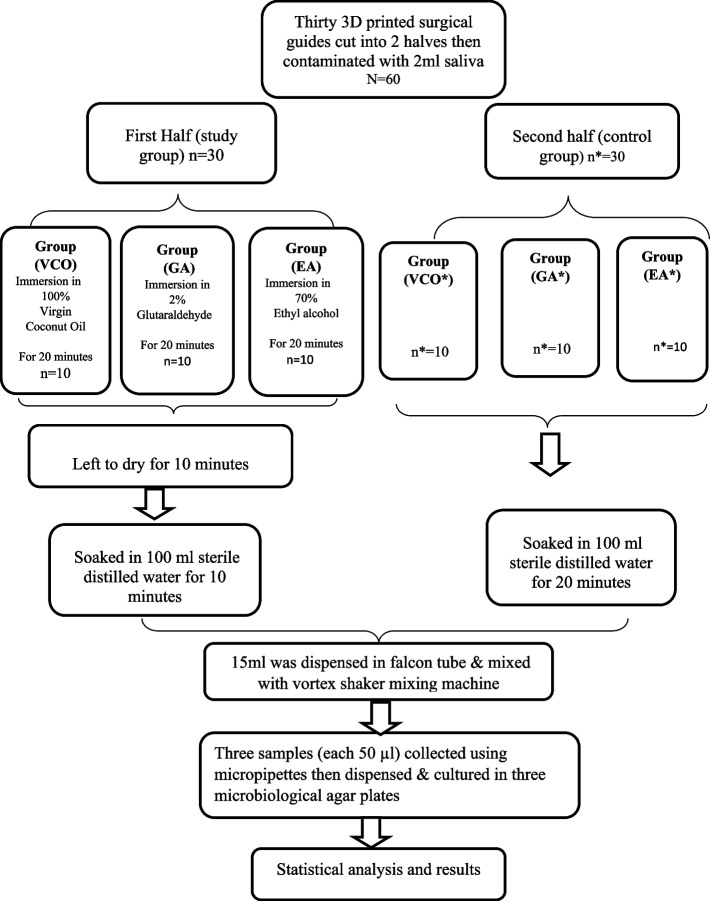
Flow chart of the study design

Thirty identical surgical guides were printed and cut into two halves (N = 60) then contaminated with a defined amount of human saliva samples (2 ml), collected from healthy partially edentulous participants attending the Department of Prosthodontics, Faculty of Dentistry, Alexandria University. The first half (n = 30) was sub-grouped into three study groups as follows: group VCO (n = 10), group GA (n = 10), and group EA (n = 10) which was immersed for 20 min in one of three disinfectants which were, 100%VCO, 2% GA, and 70% EA, then soaked in sterile distilled water. The second half (n* = 30) was sub-grouped into three control groups as follows: group VCO*(n* = 10), group GA*(n* = 10), and group EA*(n* = 10) which was soaked in sterile distilled water for 20 min for the assessment of the antimicrobial potential of the three tested disinfectants (Fig. 1).

### Surgical guides production

**Fig. 2:**
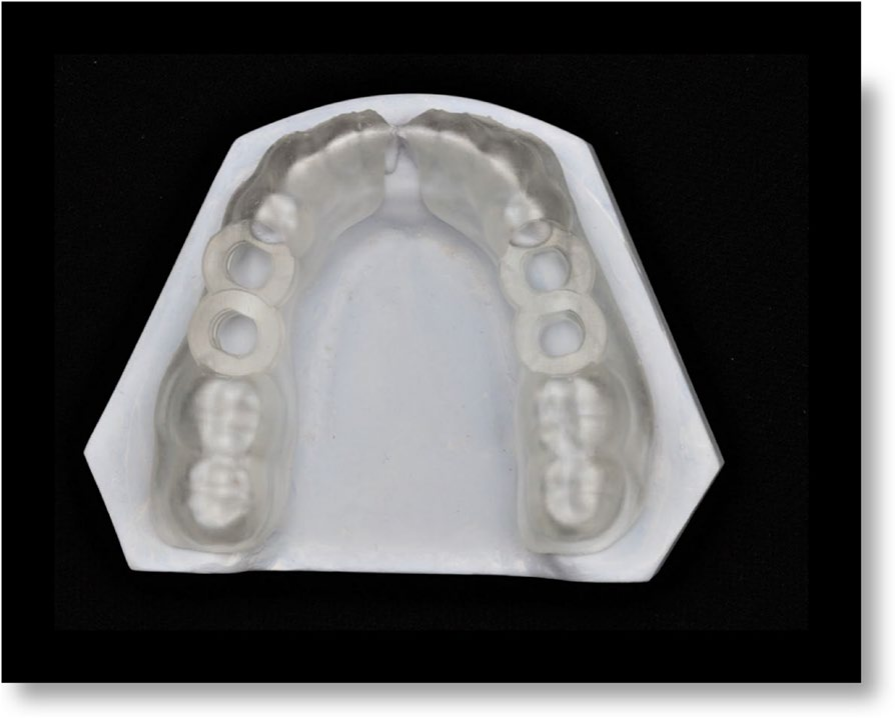
Tooth supported surgical guide designed with bilateral missing second premolars and first molars placed on a study cast

**Fig. 3:**
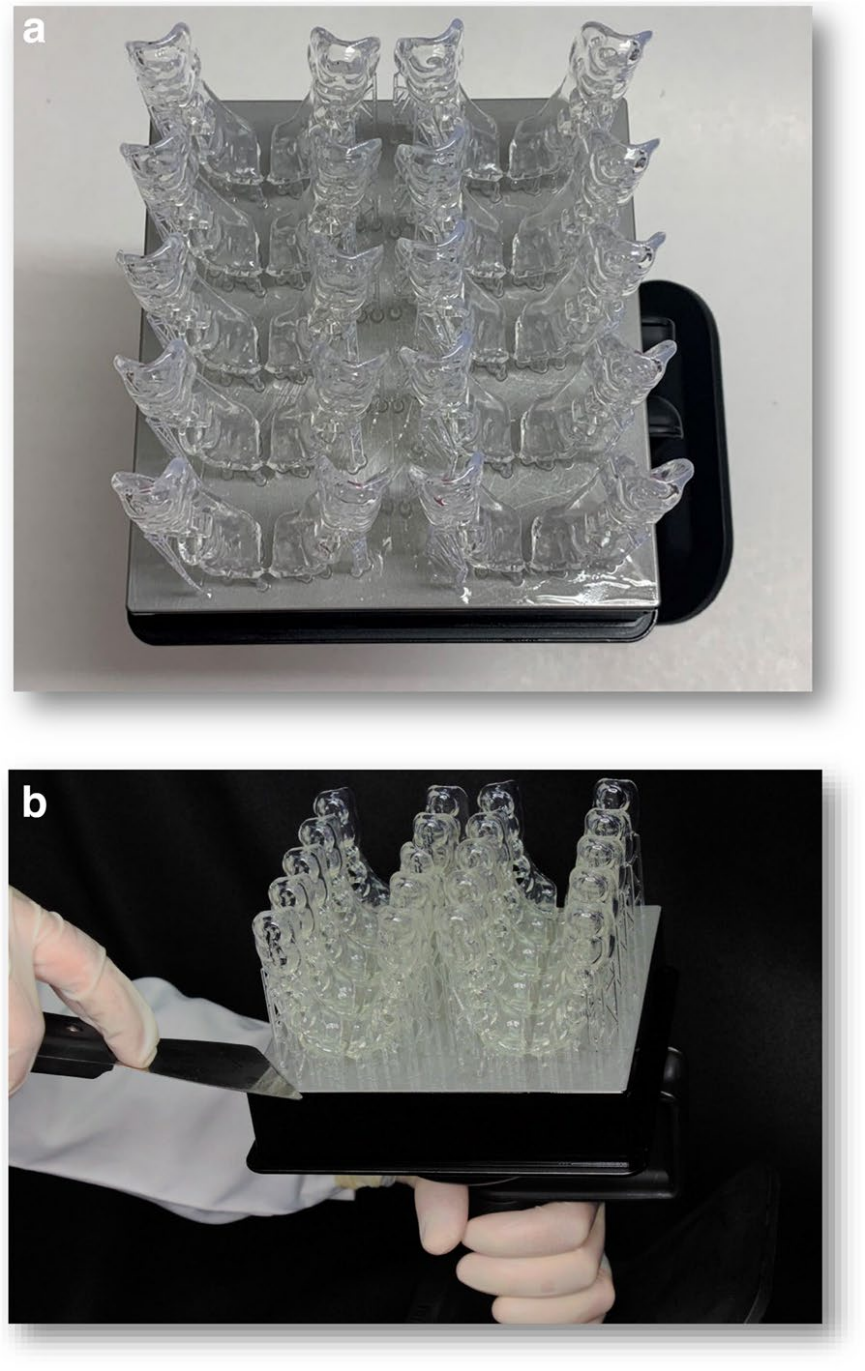
**a** Ten identical surgical guides were printed using a clear photoreactive resin material **b** 3D-printed surgical guides were removed from the build platform using a removing tool

A surgical guide was fabricated by making an impression of a mandibular dental arch with bilateral missing second premolars and first molars using polyether impression material and poured with dental stone to obtain a study cast. The study cast was scanned using CBCT (Acteon, X-mind Trium, Italy). The DICOM (Digital Imaging and Communications in Medicine) data were exported as a Standard Tessellation Language (STL) file to create a 3D model where the surgical guide was virtually designed using an open platform software (Blue Sky). Ten identical surgical guides were printed for each group using a clear photoreactive resin material (Resin cartridge form2; Formlabs Inc) using a desktop stereolithography 3D printer (formlabs 2, Formlabs Inc, Somerville, MA, USA) with the following parameters; layer thickness = 0.1 mm, layers number = 672, layer volume = 103.74 ml, offset (block out undercuts offset) = 0.15 and printing time = 6 h 45 min [33]. The 3D-printing works by adding layers of curable liquid photopolymer onto a build tray where fine layers accumulated to create 3D-surgical guides which were rinsed in a bath of 90% Isopropyl Alcohol for 10 min and then inserted in a bath of clean, unused, 90% Isopropyl Alcohol. The printed guides were left to dry for an additional 10 min and then exposed to 72 watts of Blue Ultraviolet light oven (315- 400 nm) for 10 min at 60 °C according to the manufacturer’s instruction, to achieve optimal mechanical properties. The support material was removed after curing using the flush cutter included in the Formlabs Standard Finish Kit. The surgical guides were then cut into two halves using a sterile cutting disk at low speed [34, 35] (Figs. 2 and 3a, b).

### Collection of saliva samples and contamination protocol

Prior to the start of the microbiological trial, samples of saliva were obtained from healthy partially edentulous participants. Additionally, all test subjects were clear of any type of drugs/medicines in their systems and, attending the Department of Prosthodontics, Faculty of Dentistry, Alexandria University. The saliva samples were collected directly from the human mouths by instructing the participants to spit into sterile containers in the morning before breakfast and the surgical guides were then placed inside the containers. 2 ml of fresh saliva samples were applied on the surfaces of each half using a micropipette, and the containers were then manually shaken vigorously to facilitate the dispersion of the microorganisms [36].

### Microbiological trial

**Fig. 4:**
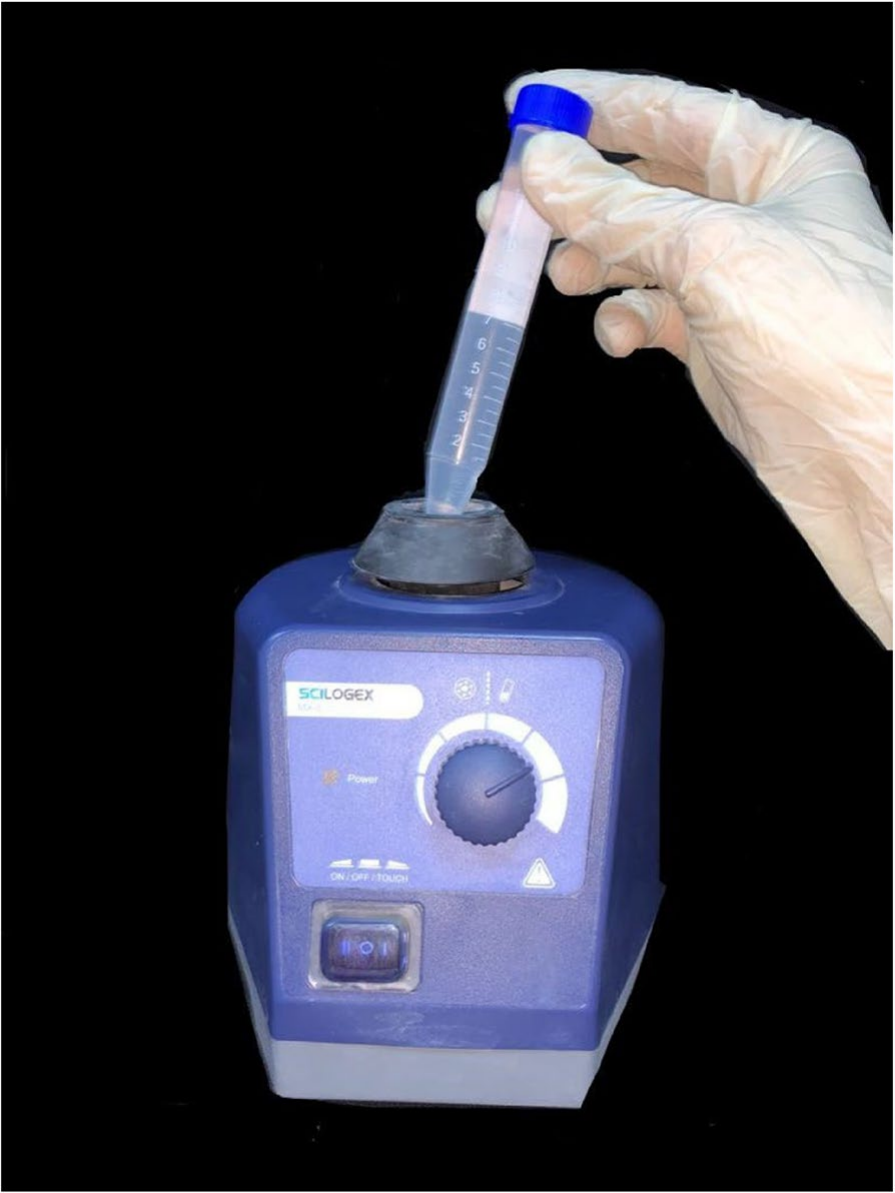
15 ml Falcon tube filled with sterile distilled water and placed on the Scilogex Vortex Mixer

After saliva contamination, the first half of the three study groups were immersed into one of the three disinfectants for 20 min then left to dry for an additional 10 min then soaked in sterile glass containers containing 100 ml. of sterile distilled water for 10 min. An extra step was done, in which a 50 µl (microliter) sample was taken directly from the surface of the surgical guides where the saliva was applied and disinfected with 100% VCO before its immersion in the sterile distilled water [37–39]. The second half of the three control groups were immersed in sterile glass containers containing 100 ml. of sterile distilled water for 20 min. From each half, a 15 ml falcon tube was filled and placed on the Scilogex Vortex mixer for 10 s (Fig. 4). Three samples were pipetted and cultured on three microbiological media; Blood, MacConkey, and Sabouraud dextrose agar plates then the microbial count was expressed as colony-forming units per plate (CFU/plate). The percentage (%) of reduction was calculated by the following equation: [40].

$$\%\;\mathrm{of}\;\mathrm{reduction}\:=\:\mathrm{Number}\;\mathrm{of}\;\mathrm{CFU}/\mathrm{control}\;\mathrm{plate}-\mathrm{CFU}/\mathrm{study}\;\mathrm{plate}/\mathrm{Number}\;\mathrm{of}\;\mathrm{CFU}/\mathrm{control}\;\mathrm{plate}\;\times100$$

### Statistical analysis

The comparison of the antimicrobial potential of the three tested disinfectants between the three study and three control groups was analyzed using the One-Way ANOVA (Analysis of variance) test that was used to verify the normality for all variables using descriptive statistics, plots, and normality tests. All variables showed normal distribution, so the mean, standard deviation (SD), and CFU/plate were calculated. The level of statistical significance was set at *p* < 0.05. Data were analyzed with IBM SPSS statistical software V 23.0, (SPSS Inc).

## Results

**Fig. 5:**
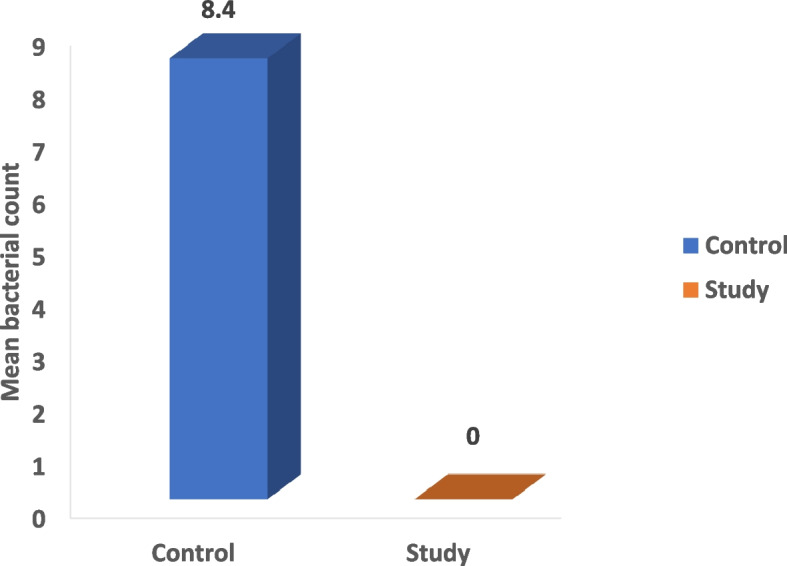
The mean bacterial count of the two halves of the surgical guides between the three control and three study groups

**Fig. 6:**
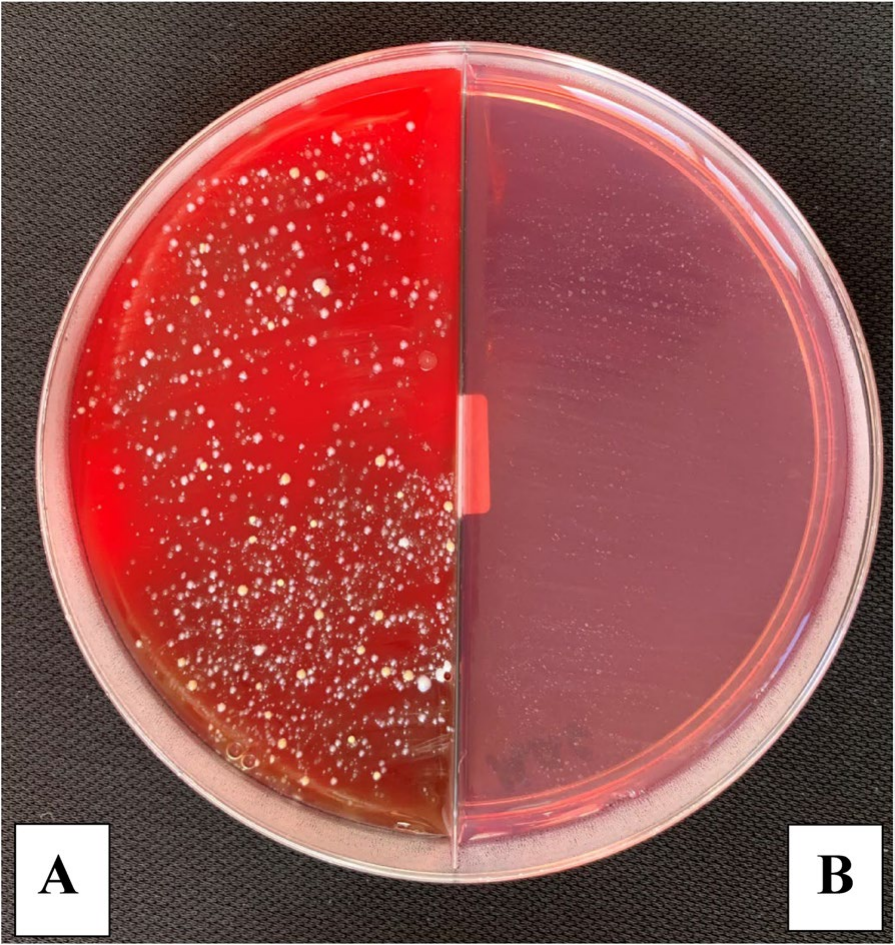
Culture results from the control group on Blood (**A**) and MacConkey (**B**) agar plate

**Fig. 7:**
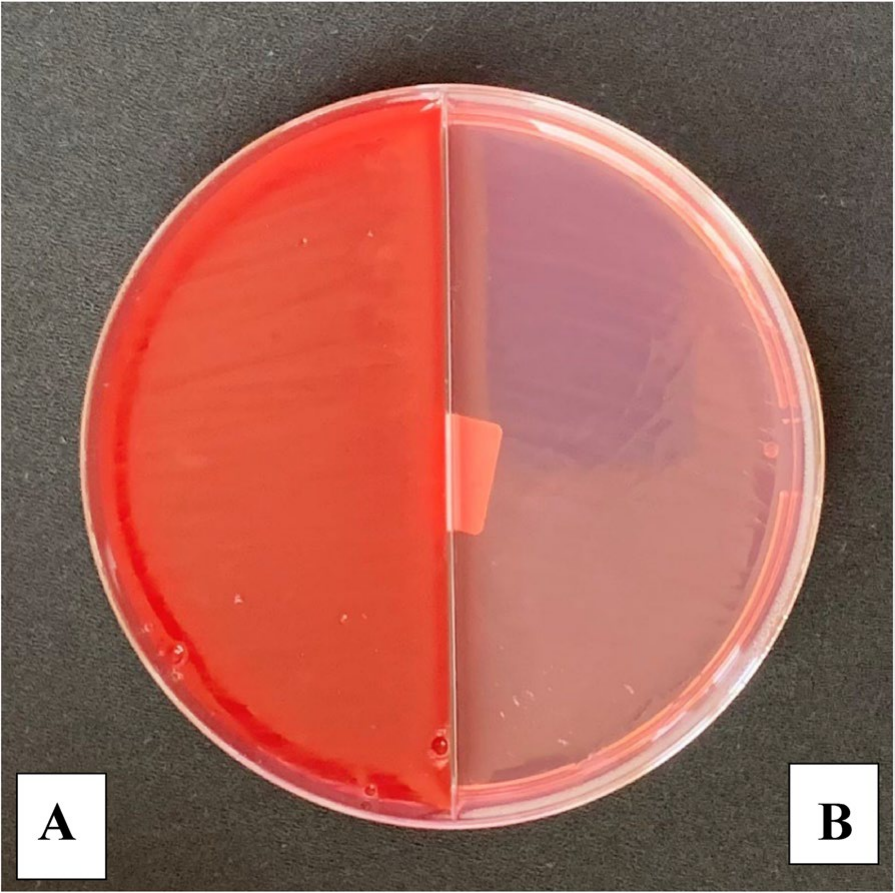
Culture results from the study group disinfected with 2% GA on Blood (**A**) and MacConkey (**B**) agar plate

**Fig. 8:**
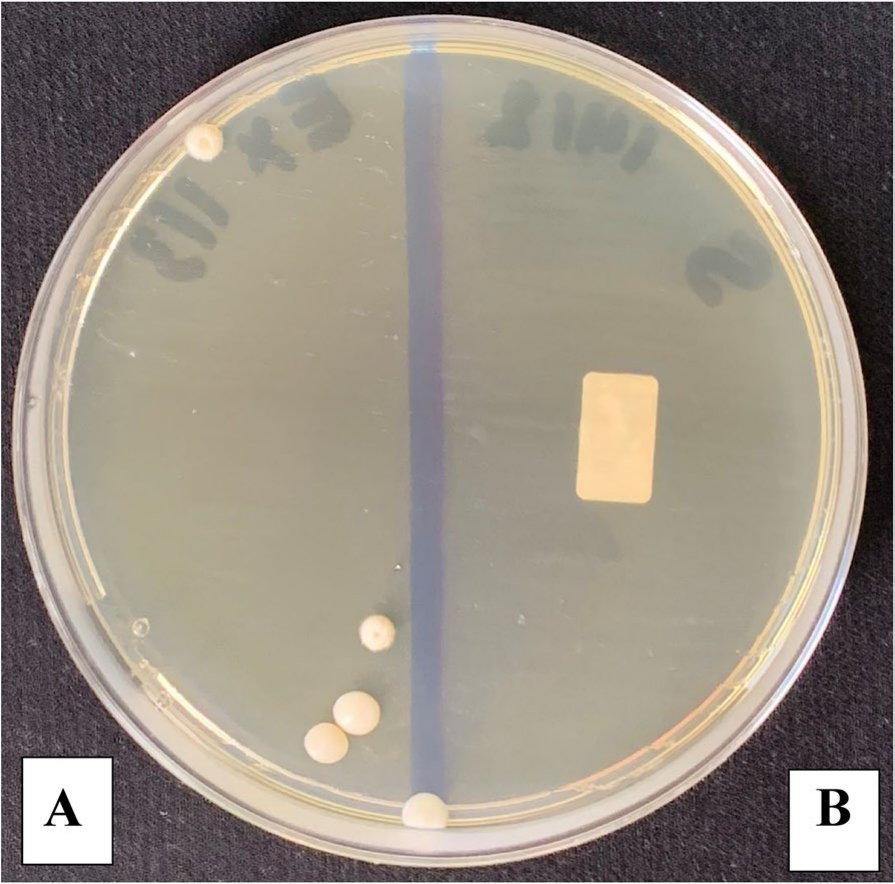
Culture results from the control (**A**) and the study group disinfected with 70% EA (**B**) on Sabouraud agar plate

**Fig. 9:**
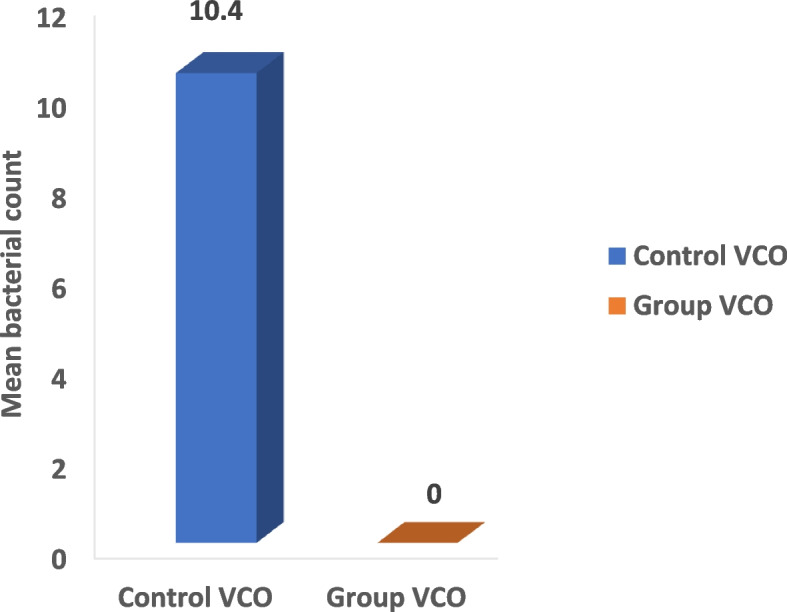
The mean bacterial count after taking a direct sample from the first half of the surgical guides disinfected with 100% VCO

**Fig. 10:**
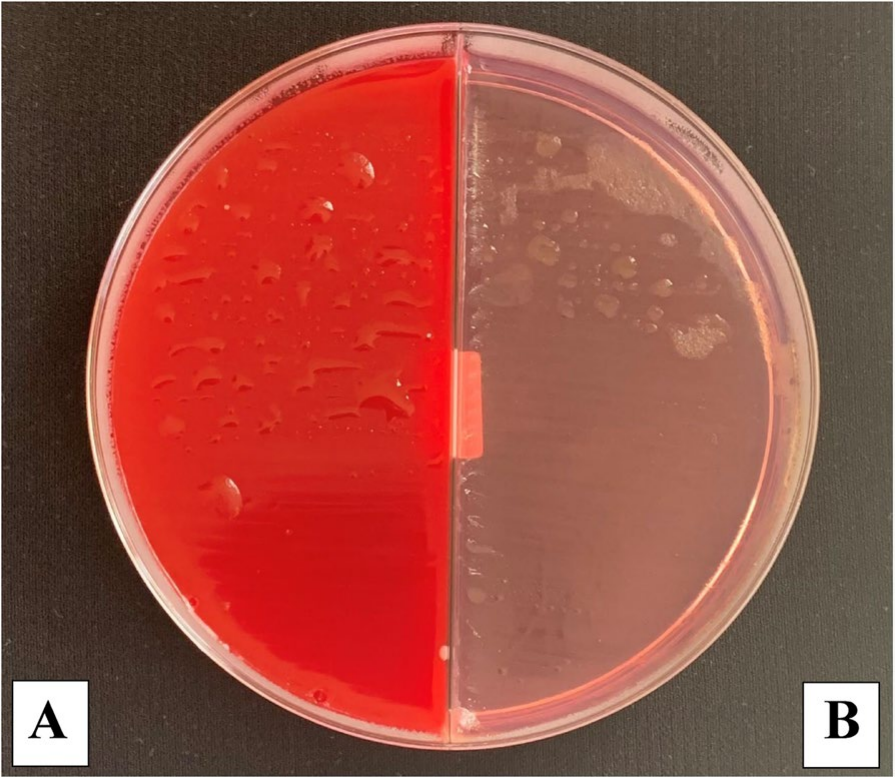
Culture results of the direct sample taken from the study group disinfected with 100% VCO on Blood (**A**) and MacConkey (**B**) agar plate

**Fig. 11:**
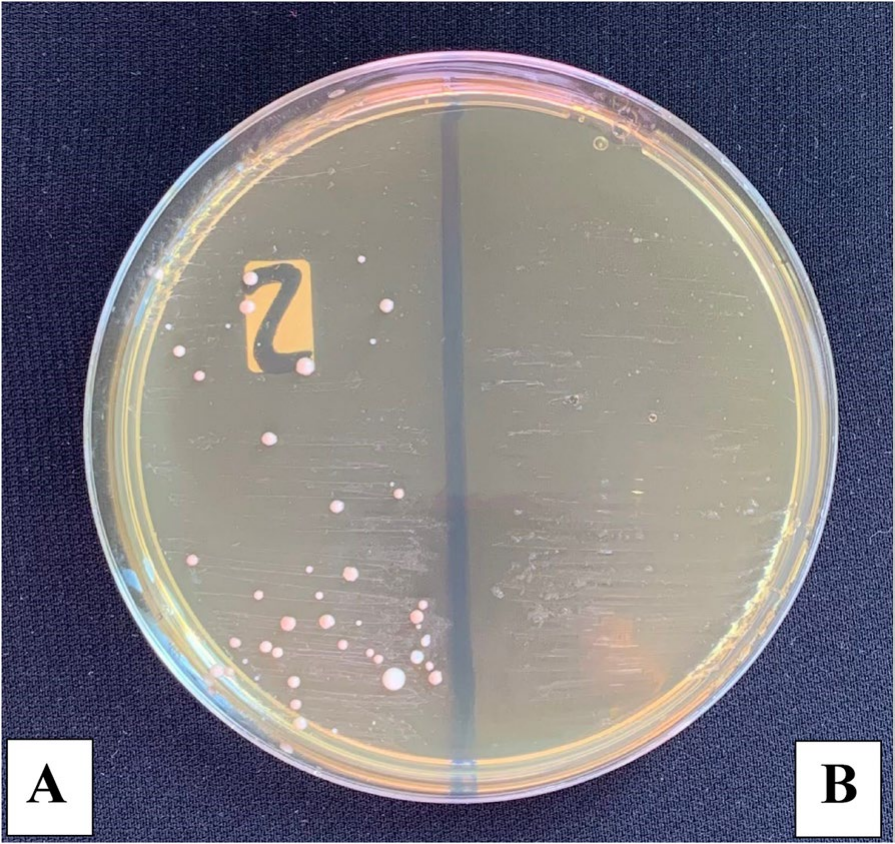
Culture results from the control (**A**) and the study group disinfected with 100% VCO (**B**) on Sabouraud agar plate

The microbial count showed statistically significant differences between the three control and three study groups (*P* < 0.001) with the highest % of reduction in the mean number of oral microorganisms (about 100%) between the three study groups. The culture results showed an uncountable bacterial growth on the control plates (more than 100 CFU/ plate) representing the baseline of the oral microorganisms and the study plates showed no bacterial growth after the use of the three disinfectants (Tables 2, 3, 4 and Figs. 5, 6, 7 and 8). Regarding the direct sample that was collected from the surface of the surgical guides disinfected with 100% VCO, no bacterial growth was revealed (Table 5, Figs. 9, 10 and 11).

**Table 2:** Mean, standard deviation (SD) and the results of comparison of CFU counts between the three control and three study groups after the addition of saliva to the two halves of the surgical guides

Three types of culturing media (Agar plates)		Control Groups (VCO^*^, GA^*^, EA^*^)	Study Groups (VCO, GA, EA)
**Blood agar**		Uncountable (> 100)	0
**MacConkey agar**		Uncountable (> 100)	0
**Sabouraud dextrose agar**	**Mean ± SD**	8.4 ± 9.02	0
	**Median (IQR)**	4.5 (2.5, 8)	
***P* value**		< 0.001**	

*IQR* Interquartile range

^**^Statistically significant *p* < .05

^*^The second half of the three control groups

**Table 3:** Mean, standard deviation (SD) and the results of comparison of CFU counts between the control and the study groups disinfected with 2% GA

Three types of culturing media (Agar plates)		Control Group (GA*)	Study Group (GA)
**Blood agar**		Uncountable (> 100)	0
**MacConkey agar**		Uncountable (> 100)	0
**Sabouraud dextrose agar**	**Mean ± SD**	2.80 ± 1.32	0
	**Median (IQR)**	3.00 (1.75, 4.00)	
***P* value**		< 0.001**	

*IQR* Interquartile range

^**^Statistically significant *p* < .05

^*^The second half of the GA control group

**Table 4:** Mean, standard deviation (SD) and the results of comparison of CFU counts between the control and the study groups disinfected with 70% EA

**Three types of culturing media (Agar plates)**		**Control Group** (EA*)	**Study Group** **(EA)**
**Blood agar**		Uncountable (> 100)	0
**MacConkey agar**		Uncountable (> 100)	0
**Sabouraud dextrose agar**	**Mean ± SD**	3.60 ± 2.27	0
	**Median (IQR)**	3.50 (1.75, 5.25)	
***P* value**		< 0.001**	

*IQR* interquartile range

^**^Statistically significant *p* < .05

^*^The second half of the EA control group

**Table 5:** Mean, standard deviation (SD) and the results of comparison of CFU counts between the control and the study groups after taking a direct sample from the first half of the surgical guides disinfected with 100% VCO

Three types of culturing media (Agar plates)		Control Group (VCO^*^)	Study Group (VCO)
**Blood agar**		Uncountable (> 100)	0
**MacConkey agar**		Uncountable (> 100)	0
**Sabouraud dextrose agar**	**Mean ± SD**	10.4 ± 11.02	0
	**Median (IQR)**	6.5 (3.5, 10)	
***P* value**		< 0.001**	

*IQR* Interquartile range

^**^Statistically significant *p* < .05

^*^The second half of the VCO control group

## Discussion

The antimicrobial potential of the three disinfectants used to decontaminate the tested surgical guides was estimated and the null hypothesis was accepted as no significant differences were found in the antimicrobial potential of 100% VCO, compared to 2% GA and 70% EA.

The microbiological results showed that the investigated disinfectants had potentially considerable inhibitory capabilities against the oral pathogens detected in the saliva samples. The use of saliva biofilm extracted from partly edentulous subjects was required to identify the oral environment and to imitate different types of bacteria commonly prevalent in the oral cavity. Consequently, this investigation might provide the foundation for future in vivo studies evaluating the antibacterial properties of VCO as a mouthwash under preoperative surgery settings [40].

Indeed, the used concentration of VCO used in the present study was 100% because researchers were having difficulty dissolving or diluting the VCO during the microbiological trial. Organic solvents commonly used to dilute VCO include methanol, ethanol, and dimethyl sulfoxide. Nonetheless, because these solvents contain antibacterial capabilities, the test results are erroneous. Until recently, the majority of antimicrobial tests were conducted directly between the tested bacteria and the extract [12]. Consequently, these findings were supported by Gayatri et al. who revealed that the concentration of the antibacterial compounds in killing bacteria is linked with the effect caused. This correlation might be described using the hyperbolic curve; thus, increasing the concentration would raise the maximal effect of the agent [41].

The immersion technique was used as a disinfection method rather than spraying as spraying reduced disinfection effectiveness because the disinfectant could not reach the entire surface, especially given the porous nature of the surgical guide surface, where microorganisms could penetrate and survive [42]. On the contrary, immersion is regarded as the most reliable and safest procedure since disinfectants were applied to all surfaces of the surgical guide [43].

It is important to highlight that the contact time for the tested disinfectants was set to be 20 min as recommended by the fabricant/ manufacturer for an ideal disinfection outcome. As a result, the CDC considers 20 min at room temperature to be the minimal exposure period required to reliably kill bacteria with 2% GA and similarly, to disinfect contaminated surfaces with 70% EA. Moreover, VCO was found to decrease the total oral microbial count, after about 20 min of contact time [44].

The use of bench-top laboratory vortex mixers during the microbiological trial was proposed since it is a method often utilized in biofilm investigations and is more commonly employed for bacterial aggregation dispersion. Because the centrifugal rotator force was the main working mechanism of a vortex mixer, it minimized the time required for mixing and the results were instant. As a result, vortexing for 10 s or more reduced bacterial viability because it acted through typical tourbillon and swirling movements and by the effect of the water flow motion, removing or reducing the adherence of microorganisms on a surface, allowing proper shaking and mixing as well as a homogeneous distribution of the microorganisms within the solution [45, 46].

The extra step that was done where a direct sample was collected from the surfaces of the first half of the surgical guides that were disinfected with 100% VCO, was extremely essential to assess the local antimicrobial effect of VCO on the oral pathogens that would be present in the oral cavity (in vivo) and to evaluate its inhibitory action on various microorganisms found in the saliva samples. Moreover, the beneficial topical effect of VCO showed promising results by eradicating 100% of the microbes found on the surgical guides. These findings were coincided with the results of other investigations that indicated the local oral effect of VCO when used during the oil-pulling therapy – an alternate, substantial, cleansing method consisting of rinsing the oral cavity by ingesting a tablespoon of VCO for 20 min of contact time – to reduce the oral bacteria and improve oral health [37–39].

Comparatively, Menaka et al. [47] confirmed the findings of this study by illustrating that VCO oil pulling was as beneficial as Chlorhexidine mouthwash in decreasing plaque. It was assumed that because of agitation, VCO oil pulling generated mechanical shear load, resulting in oil emulsification and greater surface area, which inhibited plaque adherence and microbial coaggregation. Despite being commonly regarded as the "gold standard" among other orally administered treatments, chlorhexidine-containing mouthwash had a disagreeable flavor and unfavorable adverse effects such as tooth discoloration as well as mucosal irritation [48]. A randomized clinical trial conducted by Patel et al. [49] stated that oil pulling with VCO could be used as a preventive and therapeutic agent in gingivitis due to the significant reduction in values of plaque and gingival scores in patients with mild to moderate gingivitis. Besides that, a meta-analysis suggested by Peng et al. [24] indicated that oil pulling significantly reduced oral bacterial colony count compared to water or Chlorhexidine. Dewi et al. [50] recommended that the use of VCO could show a reduction in the number of *Porphyromonas gingivalis* and *Treponema denticolaon* at the margin of the porcelain-fused-metal crown.

The results were also in agreement with Ogboulu et al. [51] who reported VCO's antifungal activity was compared to fluconazole, a first-line treatment for drug-resistant *Candida albicans*. Also, VCO and its most active fatty acids were tested in vitro for their antibacterial properties against *Clostridium difficile* [52]. Subsequently, Abbas et al. [53] investigated the antimicrobial activity of VCO and affirmed that the powerful lauric acid was highly effective against *Staphylococcus aureus, Streptococci,* and *Lactobacilli.* Similarly, the current study was comparable to Widianingrum et al. [54] who concluded that the VCO has the potential to suppress the growth of *Staphylococcus aureus* and boost the ability of phagocytic immune cells to fight it, making it a viable alternative to antibiotics and a modulator of the cellular immune system. Interestingly, Horas et al. [55] showed that applying VCO to the palatal surgical wound during the palatoplasty hastened wound healing, raised the number/amount of fibroblast cells that developed in the wound, and reduced pain sensations. Research conducted by Silalahi et al. [56] proved that applying VCO topically to wounds obtained faster healing due to the decreased epithelialization time, increased fibroblast proliferation, and higher collagen turnover. Remarkably, Khalil et al. [8] documented that when 3D-printed surgical guides were examined at various time intervals from the manufacturing stage, the antibacterial efficiency of VCO was similar to 2% GA and 70% EA without displaying any microbial growth on the tested surgical guides. Another study was performed by Kamalaldin et al. [57] on a rabbit model of allergic asthma, where the effect of VCO inhalation on airway remodeling was studied. The percentage of inflammatory cells infiltrated, the thickness of the epithelium and mucosa regions, and the quantity of goblet and proliferative cells were all reduced, indicating that VCO inhalation was successful at alleviating airway inflammation and relieving asthma-related symptoms. Along with that, Luiz Henrique C. Vasconcelos et al. [58] demonstrated for the first time that VCO supplementation had a potential role in the adjuvant treatment for guinea pigs with persistent allergic lung inflammation, due to its impact on the inflammatory and oxidative processes of the airways. Moreover, Dayrit et al. [59] provided a scientific motivation for the adoption of VCO as prospective prophylactic therapy for COVID-19 patients and a general preventive medication against numerous microbial illnesses. However, it is crucial to note that the implementation of VCO as a disinfectant was not commonly discussed in dentistry, this is why its microbiological efficiency was compared with 2% GA and 70% EA, which are the most widely used disinfectants in the dental profession [42]. Although the current study lauded the disinfectants' antimicrobial efficiency in removing 100% of the organisms detected on the contaminated surgical guides, there were concerns regarding the occupational and environmental risks generated by GA and EA [8]. Shi et al. [60] identified latent GA to be a possible mutagen, inducing significant carcinogenic effects in mice lymphocytes. Furthermore, after 15 s of contact, SARS-CoV-2 could not be fully inactivated by EA in vitro research [61]. According to research, when administered to the skin, EA had no discernible long-term residual activity; nevertheless, the regeneration of bacteria happened slowly due to the devastating impact that EA might have on the persistent microorganisms [62]. In light of the aforementioned, the present work showed that VCO had completely eradicated many oral microorganisms and could function as an effective antimicrobial agent without showing adverse effects therefore, its risk-free application together with no documented or observed reports of harmful or toxic effects had expanded the possibilities of its use against different types of infections. Hence, further studies are required to prove that VCO is highly recommended and strongly desirable not only for the disinfection of 3D-printed objects but also as a surgical site disinfectant for providing a proper sterile operative area and thus, preventing infections during the surgical procedure [40]. However, the current research has some limitations. This study focused on the microbial count together with the presence/absence of the microbial species and did not focus on the identification of particular structural properties of the bacterial characteristics present on the surface of the surgical guides prior to and following the use of the antimicrobial agents. Thus, it could be interesting to discuss an atomic force microscopy study for evaluating guides surface before and after disinfection.

## Conclusion

Based on the findings of this in vitro study, the antimicrobial potential of 100% VCO was comparable and equivalent to 2% GA and 70% EA with a significant inhibitory action against oral pathogens.

## Data Availability

The datasets used and/or analyzed during the current study are available from the corresponding author upon reasonable request.

## Abbreviations

HIV: Human Immunodeficiency Virus
SARS-COV-2: Severe Acute Respiratory Syndrome Coronavirus
3D: Three-dimensional
FDA: Food and Drug Administration
CDC: Center for Disease Control and Prevention
ADA: American Dental Association
GA: Glutaraldehyde
EA: Ethyl Alcohol
MCT: Medium Chain Triglyceride
VCO: Virgin Coconut Oil
µl: Microliter
ANOVA: Analysis of variance
CFU: Colony-forming units
SD: Standard deviation
